# Solar urticaria: clinical characteristics, treatment effectiveness, long-term prognosis, and QOL status in 29 patients

**DOI:** 10.3389/fmed.2024.1328765

**Published:** 2024-02-16

**Authors:** Shinya Imamura, Yoshiko Oda, Takeshi Fukumoto, Mayuko Mizuno, Mariko Suzuki, Ken Washio, Chikako Nishigori, Atsushi Fukunaga

**Affiliations:** ^1^Division of Dermatology, Department of Internal Related, Kobe University Graduate School of Medicine, Kobe, Japan; ^2^Department of Dermatology, Kobe City Hospital Organization, Kobe City Medical Center West Hospital, Kobe, Japan; ^3^Department of Dermatology, Osaka Medical and Pharmaceutical University, Osaka, Japan

**Keywords:** solar urticaria, UVA, UVB, VL, MUD, IIT, rush hardening therapy, DLQI

## Abstract

**Introduction:**

Solar urticaria (SU), a relatively rare skin inflammatory and photosensitivity disease, is often resistant to standard urticaria treatment. Quality of life (QOL) among SU patients has not been extensively explored. This study was performed to clarify the clinical features and effectiveness of therapies (e.g., hardening therapy) for SU and to determine QOL among SU patients.

**Methods:**

The authors examined the characteristics, treatments, and QOL statuses of 29 Japanese SU patients using medical records and a questionnaire approach.

**Results:**

Among 29 patients, H1 antihistamine therapy (H1) was effective in 22 (75.8%) patients. H2 antihistamine therapy (H2) was effective in three of seven (42.9%) patients. Ultraviolet radiation A (UVA) hardening therapy was effective in eight of nine (88.9%) patients. Visible light (VL) hardening therapy was ineffective in three of three patients. In one patient who underwent both UVA and VL hardening therapy, only UVA hardening therapy was effective. In the questionnaire, 18 patients (90%) reported some improvement compared with disease onset (four had complete remission, six had completed treatment although mild symptoms persisted, and eight were receiving treatment with moderate symptoms), whereas two patients reported exacerbation. Patients in complete remission had a mean disease duration of 4 years, whereas patients not in remission had a mean disease duration of 8.8 years. The mean Dermatology Life Quality Index (DLQI) score for the current status was 7.4. There was a correlation between DLQI and symptom/treatment status. However, neither DLQI and action spectra nor DLQI and treatments exhibited significant differences.

**Discussion:**

The questionnaire revealed current QOL status and long-term prognosis in SU patients. Compared with disease onset, most patients showed improvement when assessed for this study. Both H1 and H2 should be attempted for all SU patients. UVA hardening therapy may be an option for SU patients with an action spectrum that includes UVA.

## Introduction

Solar urticaria (SU), a relatively rare photosensitivity disease (1), is characterized by edematous erythema and wheals on areas exposed to visible light (VL) or ultraviolet radiation (UV); it can sometimes be life-threatening (2). The exposure to UV light/VL is suspected to activate a photoantigen, possibly a chromophore-derived photoproduct, that exists in the serum of affected individuals (3). Treatments for SU are limited; they include topical sunscreen, oral H1 antihistamine therapy (H1), oral H2 antihistamine therapy (H2) (4), rush hardening therapy (hardening), immunosuppressants, and biological agents (5). However, SU is often refractory to treatment (6, 7). Hardening, a standard treatment for SU, induces tolerance by repeatedly exposing a patient to gradually increasing doses of the triggering wavelengths (8). There is some evidence that ultraviolet radiation A (UVA) hardening is effective (9–11). Several hypotheses have been proposed regarding its effectiveness, such as tolerance that arises from the depletion of mast cell mediators (12), a simple photoprotective effect from delayed tanning (13), and the binding of photoallergens to IgE-binding sites (10, 14).

Only a few studies have collected epidemiological and photobiological data for SU (15, 16). Ethnic differences in skin color affect SU incidence (17, 18), and action spectra differ among ethnicities (16, 19). However, few studies have analyzed Asian patients (16, 20, 21). Furthermore, only a few studies of SU have included prognostic data (6, 19, 21) or assessed quality of life (QOL) (22–24). Thus, the long-term prognosis of SU patients, particularly with regard to QOL, is not well known.

The first aim of this study was to clarify the clinical features and effectiveness of various therapies (e.g., hardening therapy) among Japanese SU patients. The second aim was to clarify the long-term prognosis and assess QOL using a questionnaire approach. In this study, the authors analyzed data from SU patients who visited our dermatology department over a 13-year period, and the authors evaluated the results of questionnaires regarding prognosis and QOL.

## Methods

### Patient population

Twenty-nine Japanese SU patients who visited Kobe University from April 2003 to April 2016 were analyzed in this study (Table 1). Most patients had Fitzpatrick skin color type III–IV. No patients received omalizumab or immunosuppressive drugs because omalizumab was not approved during the study period; moreover, such treatments were uncommon or not covered by public insurance in Japan. All patients in this study had been diagnosed with SU on the basis of a photo-provocation test. Data regarding background characteristics and treatment effectiveness were collected from medical records. In some cases, improvement in minimal urticarial dose (MUD) was measured; in other cases, improvement in the extent of skin lesions during daily life was determined by physician interviews. These immediate post-treatment improvement data were gathered from medical records. At a few months to a few years post-treatment, all patients were mailed a questionnaire that assessed long-term improvement. The questionnaire asked patients to review their Dermatology Life Quality Index (DLQI) score for their current status. The study protocol was approved by the Institutional Review Board of Kobe University (No. 1617).

**Table 1:** Data based on the medical record.

No.	Sex	Age (y)		Complications			IgE (IU/mL)	Phototesting						Treatments		
		At first visit	At onset	Allergic rhinitis	Asthma	Atopic dermatitis		Action spectrum	Inhibition spectrum	UVA MUD (J/cm2)	VL MUD min (cm)	UVB MUD (mJ/cm2)	IIT positive with irradiated serum			
														H1 antihistamines	H2 antihistamines	Phototherapy including hardening therapy (immediate effectiveness)
1	F	56	55		(+)		223	UVA, VL	(−)	6	4 (UN)	NE	VL	Bepotastine, Cyproheptadine (partial effec.)	ND	VL (ineffec.)
2	M	25	17				NE	UVA, VL	>530 nm	8.3	10 (15 cm)	NE	(−)	Homochlorcyclizine (ineffec.)	ND	UVA (effec.), VL (ineffec.)
3	F	67	67	(+)			28	VL	>531 nm	NE	20 (30 cm)	NE	VL	Olopatadine (partial effec.)	ND	VL (ineffec.)
4	M	51	48				251	UVA, VL	(−)	8.6	20 (30 cm)	NE	VL	Olopatadine (partial effec.)	ND	VL (ineffec.)
5	F	61	60				124	UVA, UVB, VL	(−)	10	0.67 (20 cm)	169	UVA, VL	Fexofenadine (partial effec.)	ND	UVA (effec.)
6	M	23	23			(+)	NE	VL	NE	NE	40 (10 cm)	NE	NE	Loratadine (UN)	ND	ND
7	F	21	21		(+)		NE	UVA, VL	NE	12	20 (UN)	NE	NE	Fexofenadine (ineffec.)	ND	ND
8	F	57	57	(+)			322	VL	NE	NE	20 (15 cm)	NE	NE	Epinastine (partial effec.)	ND	ND
9	M	28	23				598	VL	NE	NE	30 (UN)	NE	(−)	Epinastine (partial effec.)	ND	ND
10	M	36	36				NE	VL	NE	NE	40 (UN)	NE	NE	Fexofenadine (UN)	ND	ND
11	F	6	6			(+)	51	VL	NE	NE	35 (15 cm)	NE	NE	Epinastine (partial effec.)	Details UN (ineffec.)	ND
12	M	56	56	(+)			24	UVA, VL	Latent time	8	5 (15 cm)	NE	UVA, VL	Ebastine (ineffec.)	Famotidine (ineffec.)	Oral PUVA (ineffec.)
13	F	33	33				68	UVA, VL	>540 nm	6	3 (15 cm)	NE	VL	Fexofenadine (partial effec.)	ND	UVA (effec.)
14	M	62	60	(+)			274	UVA, VL	Latent time	0.5	1 (15 cm)	NE	VL	Cetirizine (partial effec.)	ND	UVA (effec.)
15	M	17	17	(+)			308	VL	420-480 nm	NE	10 (UN)	NE	VL	Olopatadine (partial effec.)	Lafutidine (effec.)	Natural sunbathing (effec.)
16	F	27	17				NE	VL	Latent time	NE	5 (15 cm)	NE	NE	Levocetirizine (effec.)	Lafutidine (effec.)	UVA (ineffec.)
17	M	29	29				NE	VL	Latent time	NE	6 (15 cm)	NE	NE	Ebastine (partial effec.)	Lafutidine (effec.)	ND
18	M	38	35				NE	VL	Latent time	NE	15 (15 cm)	NE	NE	Bepotastine (partial effec.)	ND	ND
19	F	17	17	(+)			NE	VL	NE	NE	20 (20 cm)	NE	NE	Bepotastine (partial effec.)	ND	ND
20	M	13	11	(+)			309	VL	>450 nm	NE	2 (15 cm)	NE	VL	Olopatadine (partial effec.)	ND	ND
21	M	23	23	(+)			75	UVA, VL	NE	12	5 (30 cm)	NE	(−)	Ebastine (effec.)	Lafutidine (ineffec.)	UVA (effec.)
22	F	19	17	(+)		(+)	726	UVA, VL	NE	12	10 (20 cm)	NE	VL	Olopatadine (partial effec.)	ND	UVA (effec.)
23	F	67	67				NE	UVA, VL	Latent time	6	10 (15 cm)	NE	NE	Epinastine (partial effec.)	ND	ND
24	F	4	3				61	VL	NE	NE	3 (15 cm)	NE	(−)	Epinastine (effec.)	ND	ND
25	F	51	51		(+)		12	UVA, UVB, VL	NE	0.5	0.083 (15 cm)	120	VL	Cetirizine (ineffec.)	ND	UVA (effec.)
26	F	10	10				6	VL	NE	NE	10 (15 cm)	NE	NE	Levocetirizine (effec.)	ND	ND
27	F	22	18		(+)	(+)	214	UVA, UVB	NE	5	NE	20	NE	Fexofenadine (partial effec.)	ND	ND
28	F	51	50		(+)		653	UVA, VL	>480 nm	2	7 (15 cm)	NE	VL	Fexofenadine (UN)	ND	UVA (effec.)
29	M	49	46				NE	VL	(−)	NE	5 (30 cm)	NE	VL	Levocetirizine (effec.)	Lafutidine (ineffec.)	ND

Y, year; F, female; M, male; UVA, ultraviolet radiation A; UVB, ultraviolet radiation B; VL, visible light; MUD, minimal urticarial dose; NE, not examined; ND, not done; IIT, intradermal injection test; PUVA, psoralen-UVA; effec., effective; ineffec., ineffective.

### Photo-provocation test

Diagnoses of SU were made via photo-provocation tests that used VL, UVA, and ultraviolet radiation B (UVB) light sources. Each test used a slide projector lamp with a 250-W halogen bulb for VL (peak, 850 nm; range, 400–3,000 nm; JC24V250WS/GI; Iwasaki Electric Co. Ltd., Japan), a long-bulb fluorescent black light for UVA (peak, 350 nm; range, 310–400 nm; FL 32S; Toshiba Lighting & Technology Co., Japan), and a long-bulb fluorescent sunlamp for UVB (peak, 310 nm; range, 280–350 nm; FL 32S E-30; Toshiba Lighting & Technology Co.). The approximate flux value of narrow-band UVB (NB-UVB) was 3.9–4.5 [mW/cm^2^], and the approximate flux value of UVA was 6.7–8.1 [mW/cm^2^]. VL was not measured for slide projectors because appropriate measurement equipment was unavailable.

Photo-provocation tests with UVA/UVB were performed as previously described (10). Additionally, VL irradiation was administered on each patient’s back at a distance of 15–30 cm for 20 min. Patients who developed a wheal after exposure were diagnosed with SU and enrolled in this study; patients who developed only erythema or itchiness were diagnosed with photosensitivity and excluded from the study. Wavelengths that caused a wheal were considered the action spectra. The MUD was defined as the lowest dose that caused obvious wheals. To detect delayed reactions in some cases, skin lesions were checked at 24 and 48 h after irradiation; if a wheal appeared within a few hours or within 1 day, the latent time result was considered positive (3). As previously reported, the presence of a latent time suggested that the patient could have an inhibition spectrum (3, 16, 25).

### Inhibition spectra

To determine the inhibition spectrum, cutoff glass filters (Toshiba Medical Supply, Japan) were used to eliminate short wavelengths. After the induction of wheals by irradiation with the action spectrum, half of the wheals were shielded (3) and the exposed wheals were irradiated with wavelengths outside of the action spectrum. Wavelengths that suppressed wheal formation were regarded as the inhibition spectrum.

### Intradermal serum injection test

To detect serum photoallergens, some patients underwent an intradermal injection test (IIT) using autologous serum that had undergone *in vitro* irradiation with their action spectra. Regarding the amount of *in vitro* irradiation, there were various details. In many cases, more than MUD was irradiated to serum *in vitro*, but up to 3.3 times of MUD was irradiated *in vitro* in a case. Additionally, non-irradiated autologous serum was used as a control (26). For the IIT, 0.05 mL of serum was intradermally injected into the forearm. Natural saline was utilized as a negative control. At 15 and 30 min after injection, a wheal diameter 1.5–2.0 mm larger than the negative control was considered a positive result (27).

### Hardening therapy

The protocol for UVA hardening therapy was performed as previously described, with a maintenance dose of 10 J/cm^2^ UVA once every 1–2 weeks (9, 10). The duration varied among patients. VL hardening therapy was conducted as follows. A slide projector was used to administer VL irradiation to 3–4 parts of the body (face, abdomen, forearms, and thighs) at a distance of 10–30 cm for 3–10 min. In each subsequent treatment, VL irradiation was administered for 1 min longer than in the previous treatment. The protocol was repeated once daily for outpatients and 2–3 times per day for inpatients. The schedule was performed every day or every second day. H1 and H2 were used alone or in combination with hardening therapy.

## Results

### Patient population

**Figure 1:**
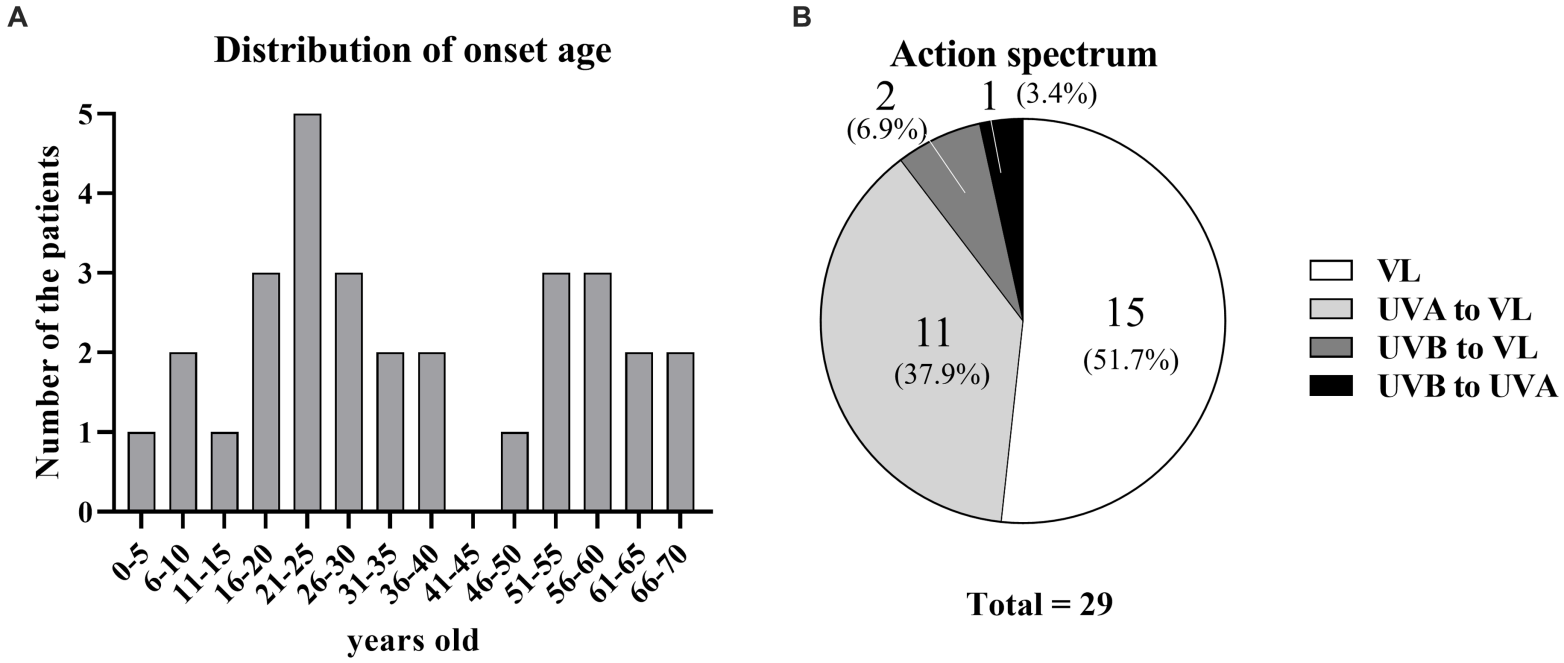
Age at onset and action spectra of SU. **(A)** Distribution of age at onset. **(B)** Percentages of action spectra.

The median patient age at the first visit was 29 years (interquartile range [IQR]: 21–51 years); there were 13 men (44.8%) and 16 women (55.2%). Sixteen patients had a history of allergic disease (eight allergic rhinitis, four asthma, two atopic dermatitis, one each allergic rhinitis and atopic dermatitis, and one each asthma and atopic dermatitis). The serum total IgE level was measured in 19 patients; the median level was 213.7 IU/mL (IQR: 56.1–308.5 IU/mL). The median age at onset was 29 years (IQR: 17–51 years); it showed a bimodal distribution, with peaks at 21–25 and 51–60 years (Figure 1A). Patient characteristics are shown in Table 1.

The questionnaire response rate was 69% (20 of 29 patients) (Table 2). The median age at the time of questionnaire response was 46 years (IQR: 29–65 years). The median time from disease onset until questionnaire response was 7 years (IQR: 3.75–14 years).

**Table 2:** Data based on the questionnaires.

No.	Response to questionnaire	Age (y)			Current status		DLQI	
		At onset	At response	At complete remission	Current improvement	Current Treatment	Current status	QOL disability
1	(+)	55	69		Improve (mild symp.)	(−)	2	Mild dist.
2	(+)	17	42		Improve (mild symp.)	(−)	5	Mild dist.
3	(+)	67	80		Improve (mild symp.)	(−)	7	Moderate dist.
4	(+)	48	63		Deteriorate	UN	20	Severe dist.
5	(+)	60	77	71	Complete remission	(−)	0	No dist.
6	NA	23	NA		NA	NA	NA	NA
7	NA	21	NA		NA	NA	NA	NA
8	(+)	57	64	59	Complete remission	(−)	0	No dist.
9	(+)	23	37		Improve (moderate symp.)	Clemastine Fumarate, Fexofenadine (UAN)	16	Severe dist.
10	NA	36	NA		NA	NA	NA	NA
11	NA	6	NA		NA	NA	NA	NA
12	NA	56	NA		NA	NA	NA	NA
13	(+)	33	40	35	Complete remission	(−)	0	No dist.
14	(+)	60	69		Improve (moderate symp.)	Olopatadine	3	Mild dist.
15	NA	17	NA		NA	NA	NA	NA
16	(+)	17	31		Improve (moderate symp.)	Levocetirizine (UAN)	4	Mild dist.
17	NA	29	NA		NA	NA	NA	NA
18	(+)	35	43		Improve (mild symp.)	(−)	3	Mild dist.
19	(+)	17	22		Improve (moderate symp.)	Olopatadine	13	Severe dist.
20	(+)	11	17		Improve (mild symp.)	(−)	11	Severe dist.
21	NA	23	NA		NA	NA	NA	NA
22	(+)	17	21		Improve (mild symp.)	(−)	10	Moderate dist.
23	(+)	67	69	68	Complete remission	(−)	0	No dist.
24	(+)	3	6		Improve (moderate symp.)	Epinastine	7	Moderate dist.
25	(+)	51	53		Improve (moderate symp.)	Levocetirizine, Lafutidine, Montelukast, hardening	7	Moderate dist.
26	NA	10	NA		NA	NA	NA	NA
27	(+)	18	23		Deteriorate	Fexofenadine (UAN)	13	Severe dist.
28	(+)	50	51		Improve (moderate symp.)	Fexofenadine, hardening	17	Severe dist.
29	(+)	46	49		Improve (moderate symp.)	Loratadine, Lafutidine	10	Moderate dist.

Y, year; DLQI, Dermatology Life Quality Index; CR, complete remission; UAN, use as needed; ND, not done; UN, unknown; NA, no answer; symp, symptom; dist., disturbance. The patients who did not answer to the questionnaires were described as gray rows. The patients’ number is coincident with Table 1. The same number expresses the same patient.

### Photo-provocation test and IIT

Among the 29 patients, photo-provocation tests revealed action spectra of VL only in 15 patients (51.7%), UVA to VL in 11 patients (37.9%), UVB to VL in two patients (6.9%), and UVB to UVA in one patient (3.4%) (Figure 1B; Table 1). Because only one patient did not have an action spectrum that included VL, 28 patients (96.5%) were at least sensitive to VL.

The median MUDs were 10 min (IQR: 4.75–20) for VL, 7 J/cm^2^ (IQR: 5–10) for UVA, and 120 mJ/cm^2^ (IQR: 70–145) for UVB. An inhibition spectrum was confirmed in 6 of the 10 tested patients. Detailed examination of the wavelengths revealed an inhibition spectrum longer than 420 nm in all six patients. A latent time was detected in six patients. The IIT with autologous serum irradiated with their action spectrum *in vitro* was positive in 13 of 17 patients tested (76.5%): 11 patients were positive to irradiated serum with VL, and 2 patients were positive for irradiated serum with both VL and UVA.

### Treatment selection

All 29 patients underwent H1 (Table 1); 12 of these patients underwent H1 only. The dose of H1 was limited to a 2-fold increase because of insurance coverage-related restrictions in Japan. Because all patients attempted H1 only as the first step, the effectiveness of H1 only could be measured in all patients. Ten patients underwent both H1 and hardening therapy; three patients underwent both H1 and H2; and two patients underwent H1, H2, and hardening therapy. One patient underwent H1, H2, and oral psoralen-UVA (PUVA) therapy; one patient underwent H1, H2, and natural sunbathing therapy (the sunbathing protocols varied and are not described in this article).

### Treatment effectiveness

Treatment effectiveness was examined on the basis of medical records (i.e., without questionnaire responses) or via MUD measurements (Table 1). When the reviewing physician recorded improvement based on the patient’s subjective symptoms or the MUD was improved after treatment, the treatment was considered effective. To eliminate subjective patient data, the authors divided patients into a “MUD-based review group” and an “Interview-based review group” (Supplementary Figures S1, S2). When treatment effectiveness was adequate and urticaria did not appear in daily life, or when urticaria was not induced during a photo-provocation test, the treatment was considered “effective.” If the treatment exhibited partial effectiveness but urticaria remained present or another additional treatment was needed, or the prolonging of MUD was observed but urticaria appeared by photo-provocation test, the treatment was considered “partially effective.”

**Figure 2:**
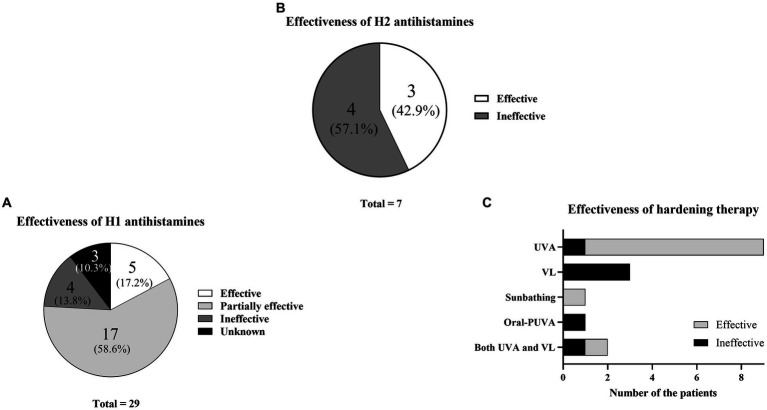
Treatment effectiveness. **(A)** Percentages of H1 antihistamine effectiveness. **(B)** Percentages of H2 antihistamine effectiveness. **(C)** Effectiveness of hardening therapy according to wavelength.

The results for H1 only were as follows: five effective (17.2%), 17 partially effective (58.6%), four ineffective (13.8%), and three unknown (10.3%) (Figure 2A; Table 1). The results for H2 only were three effective (42.9%) and four ineffective (57.1%) (Figure 2B; Table 1). Hardening with UVA was effective in eight patients and ineffective in one patient (Figure 2C; Table 1). The patient with an ineffective treatment result had an unusual situation in which the action spectrum comprised VL, but UVA hardening was attempted. Hardening with VL was ineffective in three of three patients. Natural sunbathing was effective in one patient. Oral PUVA was ineffective in one patient. In one patient who underwent hardening with both UVA and VL, UVA hardening was effective, whereas VL hardening was ineffective.

### Current symptoms according to the questionnaire

**Figure 3:**
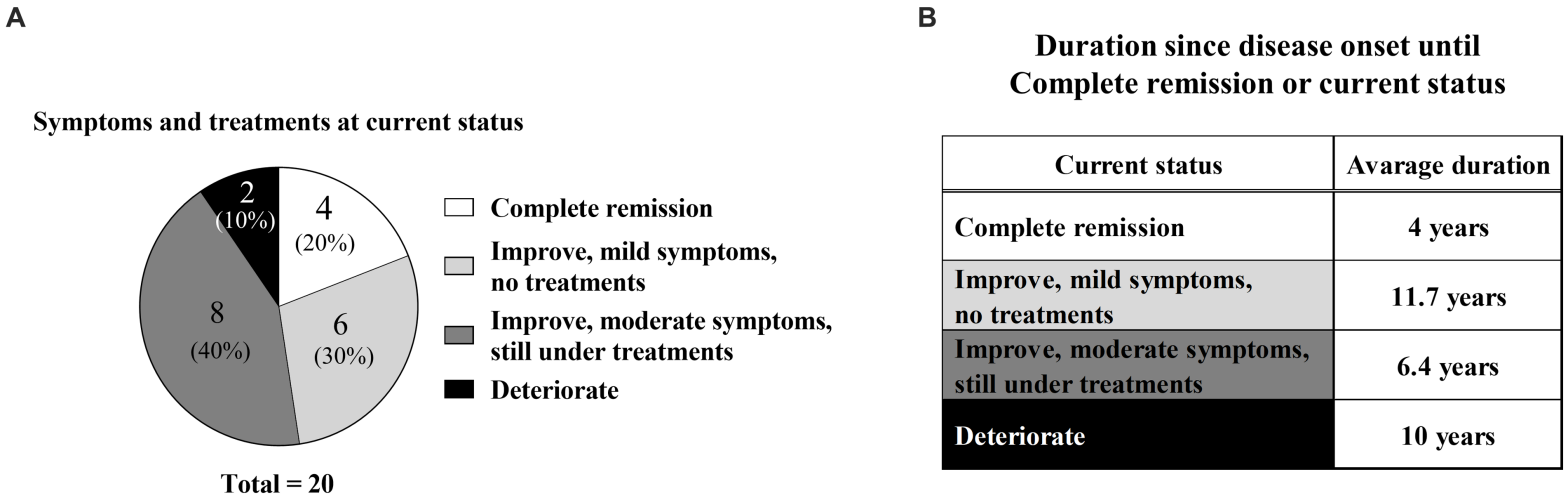
Current symptoms and time from disease onset. **(A)** Percentages of current symptoms and current treatment conditions. **(B)** Time from disease onset to complete remission or current status.

Twenty of 29 patients responded to the questionnaire. Their current statuses were as follows (Table 2; Figure 3A): complete remission in four (20%), improvement with mild symptoms but not undergoing treatment in six (30%), improvement with moderate symptoms but continuing treatment in eight (40%), and deterioration in two (10%). In particular, nine patients (45%) were continuing H1, whereas two patients (10%) were continuing hardening therapy based on their current status (Table 2).

In the complete remission group, the mean disease duration from onset to complete remission was 4 years; the specific durations for each of the four patients were 1, 2, 2, and 11 years (Figure 3B; Table 2). In contrast, the mean disease durations were 11.7 years in the improvement with mild symptoms group, 6.4 years in the improvement with moderate symptoms group, and 10 years in the deterioration group (Figure 3B). The mean disease duration in the non-complete remission group (combination of mild symptoms, moderate symptoms, and deterioration groups) was 8.8 years.

### Dermatology life quality index score for current status according to the questionnaire

**Figure 4:**
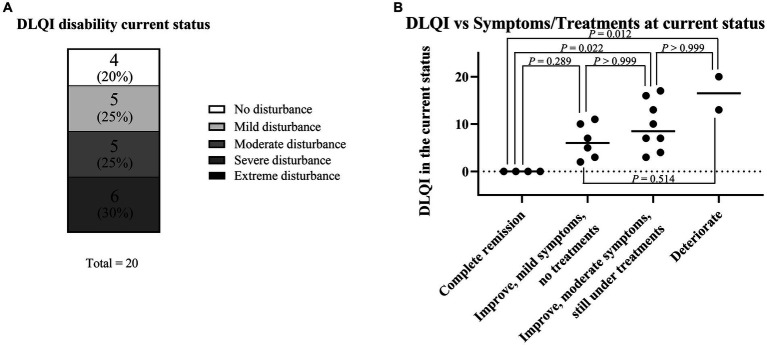
Current symptoms and comparison with DLQI score for the current status. **(A)** DLQI score for the current status. **(B)** Association between DLQI score for the current status and current symptoms/treatments. Because continuous variables showed a non-normal distribution according to the Shapiro–Wilk test and each sample was independent, statistical analyses were performed using the Kruskal–Wallis test followed by Dunn’s multiple comparisons test.

To analyze QOL status among SU patients, the authors collected DLQI data via questionnaire. The patients were classified into five groups depending on their DLQI scores to visualize the degree of QOL impairment (Figure 4A; Table 2). Briefly, DLQI scores 0–1 were classified as “no disturbance,” 2–5 as “mild disturbance,” 6–10 as “moderate disturbance,” 11–20 as “severe disturbance,” and 21–30 as “extreme disturbance” (28). Thus, four patients (20%) had “no disturbance,” five patients (25%) had “mild disturbance,” five patients (25%) had “moderate disturbance,” six patients (30%) had “severe disturbance,” and no patients had “extreme disturbance.”

The association between the DLQI score for the current status and current symptoms/treatments was analyzed (Figure 4B). The median DLQI scores in each group were as follows: complete remission, 0 (IQR: 0–0); mild symptoms, 6 (IQR: 3.5–9.25); moderate symptoms, 8.5 (IQR: 6.25–13.75); and deterioration, 16.5 (IQR: 14.75–18.25). There were significant differences in DLQI score between the complete remission and moderate symptom groups (*p* = 0.022) and between the complete remission and deterioration groups (*p* = 0.012).

### Dermatology life quality index score for current status according to the action spectrum and treatments

**Figure 5:**
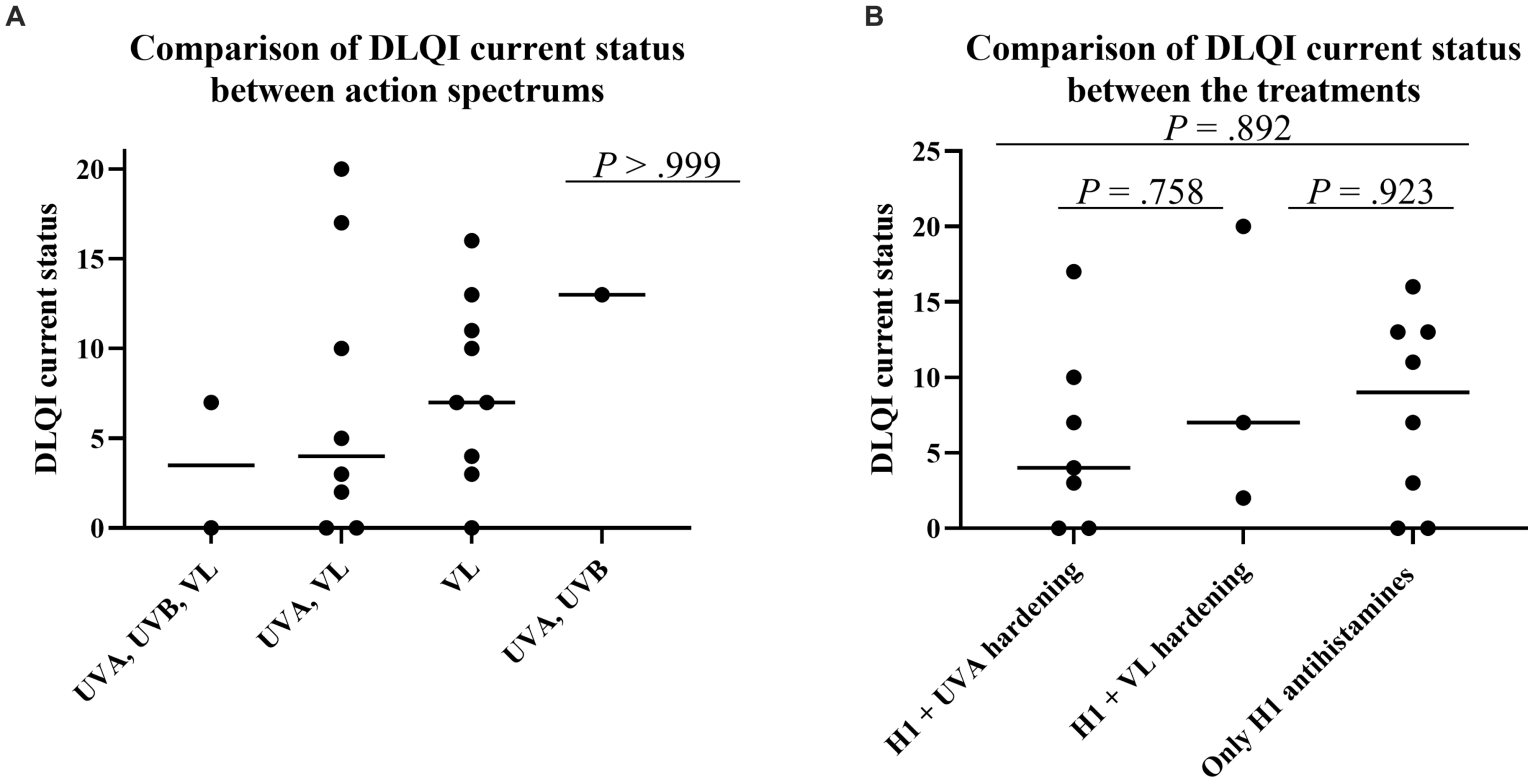
Factors affecting DLQI score for the current status. **(A)** Comparison of DLQI score for the current status according to action spectrum at onset. Because continuous variables showed a non-normal distribution according to the Shapiro–Wilk test and each sample was independent, statistical analyses were performed using the Kruskal–Wallis test followed by Dunn’s multiple comparisons test. **(B)** Comparison of DLQI score for the current status according to treatment. Because continuous variables showed a non-normal distribution according to the Shapiro–Wilk test and each sample was independent, statistical analyses were performed using the Kruskal–Wallis test followed by Dunn’s multiple comparisons test.

The authors examined factors affecting the DLQI score for the current status. The action spectrum at onset did not affect the DLQI score for the current status (Figure 5A). Similarly, treatment selection (H1, UVA hardening, and/or VL hardening) did not affect the DLQI score for the current status (Figure 5B).

In addition to the above factors, the authors found no differences in other factors (sex, age at onset, complications, total serum IgE level, inhibition spectrum, and time from disease onset).

### Statistical analysis

As shown in Figures 4B, 5A,B, continuous variables exhibited a non-normal distribution according to the Shapiro–Wilk test and each sample was independent; thus, statistical analyses were performed using the Kruskal–Wallis test followed by Dunn’s multiple comparisons test. Statistical analysis was performed with GraphPad Prism 8 software (GraphPad Software Inc., La Jolla, CA).

## Discussion

In this study, the authors used a questionnaire approach to explore long-term prognosis and QOL among SU patients. The authors examined factors affecting long-term prognosis within a few years after treatment. To our knowledge, no previous studies have compared treatment selection, immediate effectiveness based on medical records, and long-term prognosis within a few years after treatment (using a questionnaire).

Here, the authors discuss patient characteristics (Table 1). Although a previous report mentioned sex differences in SU incidence, prognostic differences were not recorded (29). In the present study, the authors analyzed sex differences; although the male-to-female ratio was 13:16, DLQI scores did not differ between men and women. Age at onset of SU was previously described as young (median ages of 32 or 35 years) (16, 29). In the present study, the median age at onset was similar but tended to be younger (29 years). Additionally, the authors found that no patient had an IgE level exceeding 1,000 IU/mL. Many patients had action spectrum to VL was also reported in another Japanese report (16). This may reflect the difference in the action spectrum between the yellow race and Caucasians.

Regarding treatment selection, H1 effectiveness has been considered insufficient compared with other treatments (6, 30, 31). However, when the authors grouped patients according to H1 effectiveness (effective/partially effective), 22 of the 29 patients (72.4%) experienced at least partial H1 effectiveness (Figure 4A). These findings suggest that H1 can be recommended as a first-line treatment. However, many patients underwent combination therapy with the addition of H1; this fact also expressed that the effectiveness of H1 was found, but it was limited and not enough (If the authors excluded the partially effective group of 55.2%, the effective group was only 17.2%) (Figure 4A).

Next, the authors analyzed treatment selection and immediate effectiveness based on medical records. Hardening therapy was selected for 17 patients. Eight of nine patients who underwent UVA hardening showed improvement, whereas three of the three patients who underwent VL hardening showed no improvement (Figure 4C). Thus, UVA hardening was considered an effective treatment in the present study. Compared with UVA hardening, very few reports have described successful VL hardening treatment outcomes (10). Nevertheless, our VL hardening protocol is experimental and requires further optimization. Thus, when an SU patient has an action spectrum that includes UVA, UVA hardening may be effective. In contrast, when the patient has an action spectrum of VL only, VL hardening is not strongly recommended; however, methods for VL hardening require further modification.

Long-term prognostic data for SU are currently limited. In the present study, when the authors combined the improvement groups (complete remission, improvement with mild symptoms, and improvement with moderate symptoms), the authors observed that 18 (90%) of 20 patients experienced some improvement (Figure 5A). This tendency may reflect a natural recovery aspect of SU as previously mentioned (19). Notably, the complete remission group comprised only four (20%) of 20 patients, with complete remission at a mean of 4 years after disease onset (Figure 5B). This percentage was similar to the percentages in another report (12% at 5 years and 26% at 10 years after diagnosis) (19). These data imply that SU is refractory in many patients; although most patients experienced at least partial recovery, complete remission was rare and challenging to achieve. Furthermore, in terms of disease duration, complete remission occurred within a mean of 4 years after disease onset. This was a shorter duration compared with the non-complete-remission group (Figure 5B). The four patients with complete remission had the following features: female sex, various action/inhibition spectra, H1 + UVA hardening in two patients, and H1 only in two patients (Table 1).

QOL status among SU patients has rarely been evaluated using DLQI scores (32). Thus, long-term prognostic assessment and DLQI measurement are strengths of this study. The authors compared DLQI scores for the current status among patients who underwent hardening therapy; the authors found that neither action spectra nor treatment selection affected the DLQI score for the current status (Figures 2A,B). Thus, although hardening therapy showed some immediate effectiveness, such therapy did not have a long-term prognostic effect on the DLQI score for the current status.

This study had some limitations. Because SU is relatively a rare disease, only 29 patients were included. This is a limitation of the single-center design; an international multi-center study is needed to increase the number of patients. Furthermore, the DLQI score reflects subjective symptoms, rather than objective indicators. Finally, because H1 was administered to all patients, the authors could not perform comparisons with a control group of SU patients who did not undergo H1 treatment. Therefore, a larger, standardized study that prospectively collects appropriate data, including baseline information, is needed. Additionally, most judgments of treatment effectiveness were made on the basis of MUD, but a few judgments of treatment effectiveness were determined on the basis of physician-mediated patient interviews; the authors highlight the differences between these groups in Supplementary Figures S1, S2.

In conclusion, our questionnaire analysis revealed long-term prognosis and current QOL status in SU patients. Most patients showed some improvement when assessed for this study, compared with disease onset. This study confirmed the effectiveness of hardening therapy, especially UVA hardening therapy for SU patients with an action spectrum that includes UVA.

## Data availability statement

The raw data supporting the conclusions of this article will be made available by the authors, without undue reservation.

## Ethics statement

The studies involving humans were approved by the Institutional Review Board of Kobe University. The studies were conducted in accordance with the local legislation and institutional requirements. Written informed consent for participation in this study was provided by the participants' legal guardians/next of kin. Written informed consent was obtained from the individual(s) for the publication of any potentially identifiable images or data included in this article.

## Author contributions

SI: Data curation, Formal analysis, Funding acquisition, Investigation, Methodology, Project administration, Software, Visualization, Writing – original draft, Writing – review & editing. YO: Conceptualization, Data curation, Investigation, Methodology, Writing – review & editing. TF: Data curation, Methodology, Writing – review & editing. MM: Data curation, Methodology, Writing – review & editing. MS: Data curation, Methodology, Writing – review & editing. KW: Conceptualization, Data curation, Methodology, Validation, Writing – review & editing. CN: Supervision, Writing – review & editing. AF: Conceptualization, Data curation, Investigation, Methodology, Supervision, Writing – original draft, Writing – review & editing.
