# Supplementary material for: Solar urticaria: clinical characteristics, treatment effectiveness, long-term prognosis, and QOL status in 29 patients
Source: Front Med (Lausanne). 2024 Feb 16;11:1328765. doi: 10.3389/fmed.2024.1328765 (PMC10904580; doi:10.3389/fmed.2024.1328765)
Supplement: Supplementary file 1 [file Presentation_1.PPTX]

## Slide 1
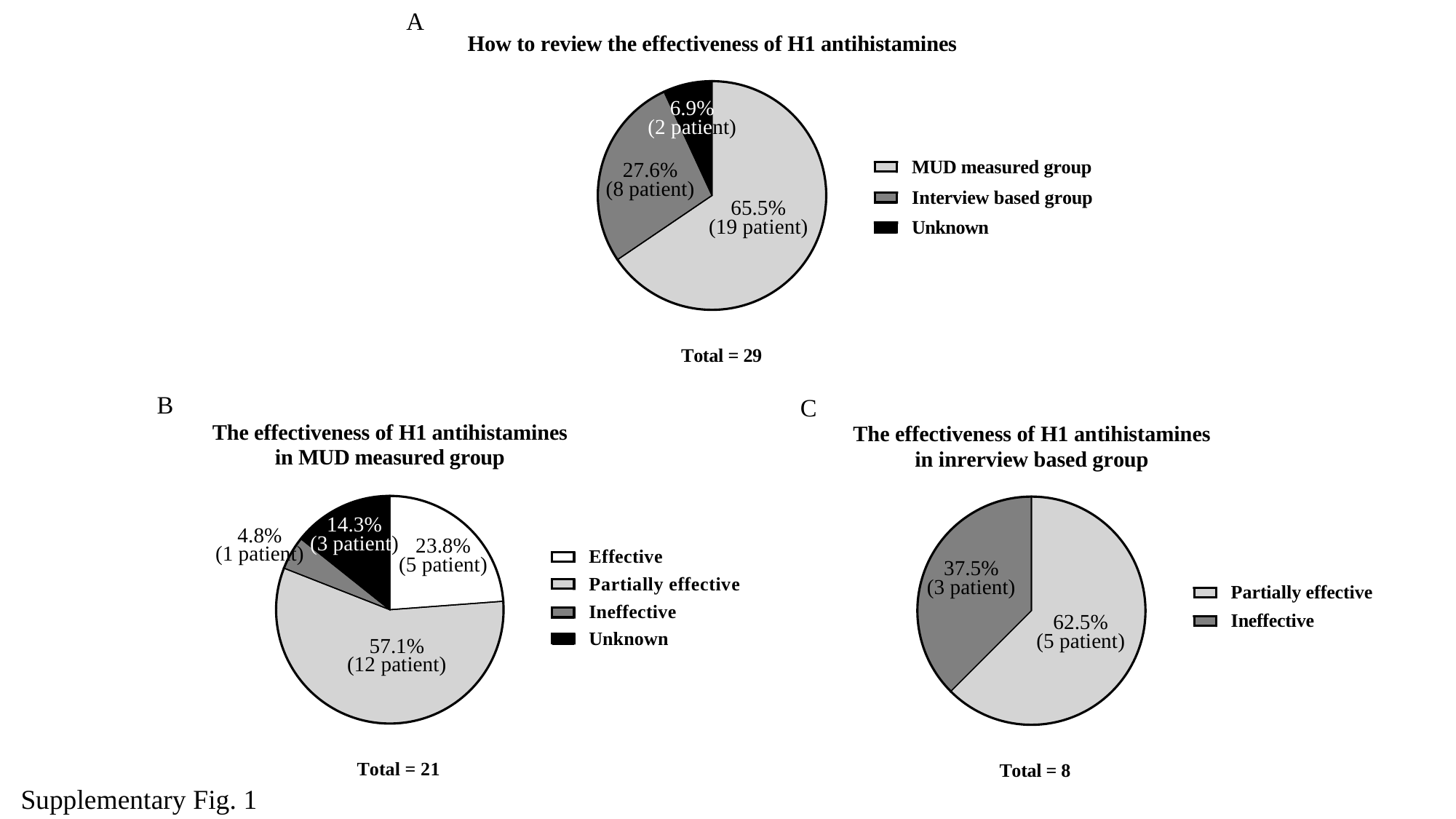

A
6.9%
(2 patient)
27.6%
(8 patient)
65.5%
(19 patient)
B
C
14.3%
(3 patient)
4.8%
(1 patient)
23.8%
(5 patient)
37.5%
(3 patient)
62.5%
(5 patient)
57.1%
(12 patient)
Supplementary Fig. 1

## Slide 2
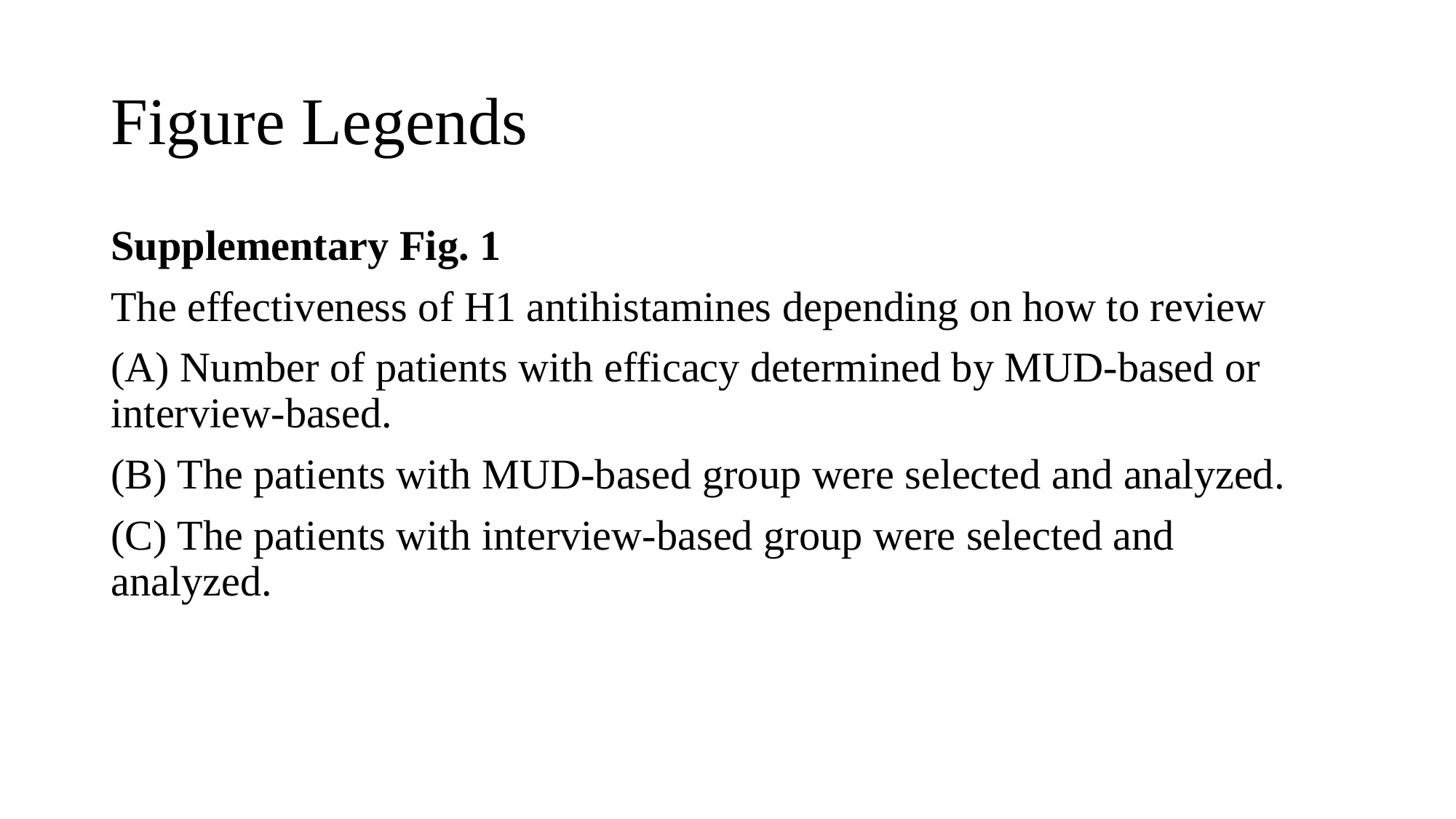

# Figure Legends
Supplementary Fig. 1
The effectiveness of H1 antihistamines depending on how to review
(A) Number of patients with efficacy determined by MUD-based or interview-based.
(B) The patients with MUD-based group were selected and analyzed.
(C) The patients with interview-based group were selected and analyzed.

## Slide 3
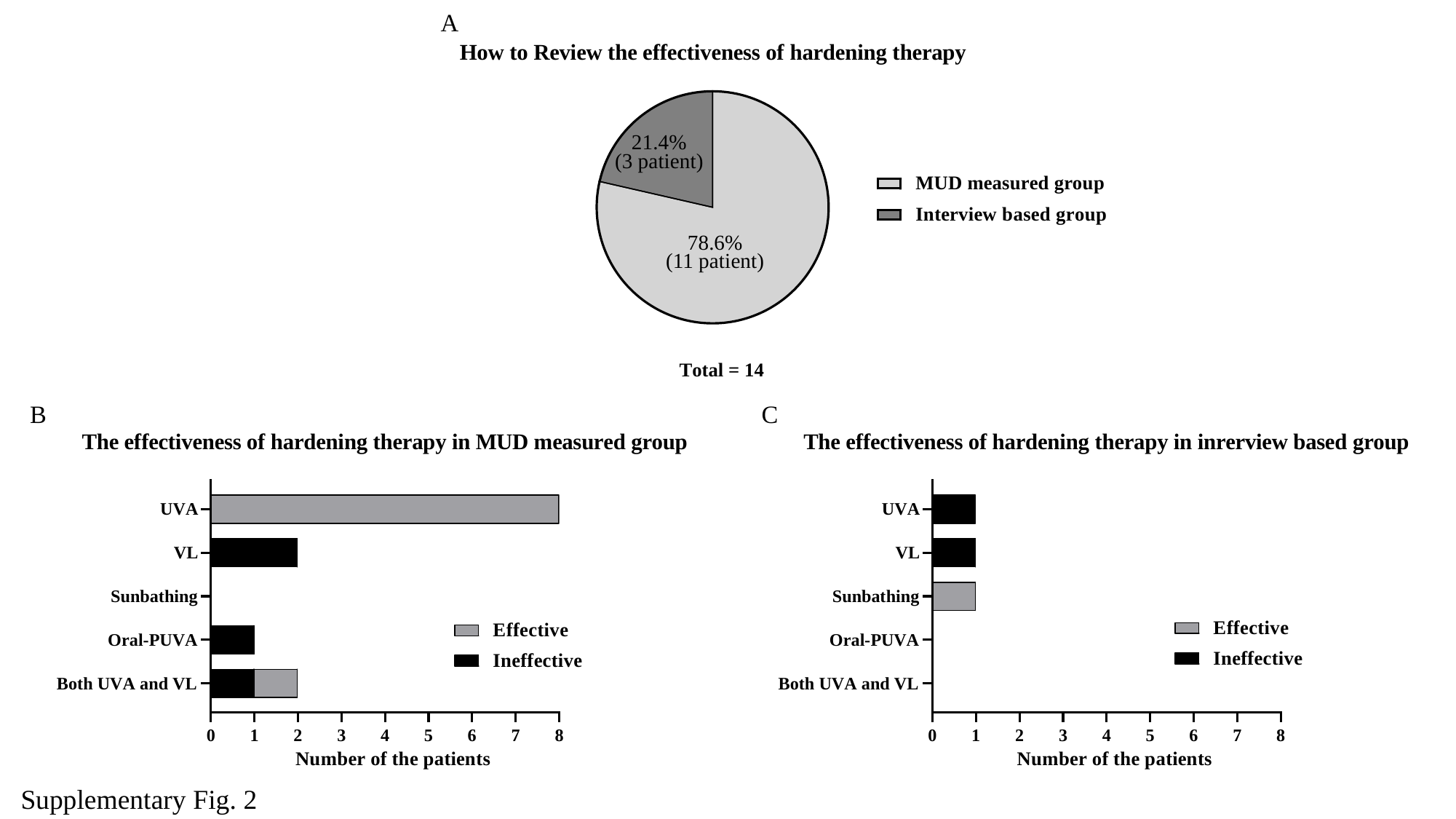

A
21.4%
(3 patient)
78.6%
(11 patient)
B
C
Supplementary Fig. 2

## Slide 4
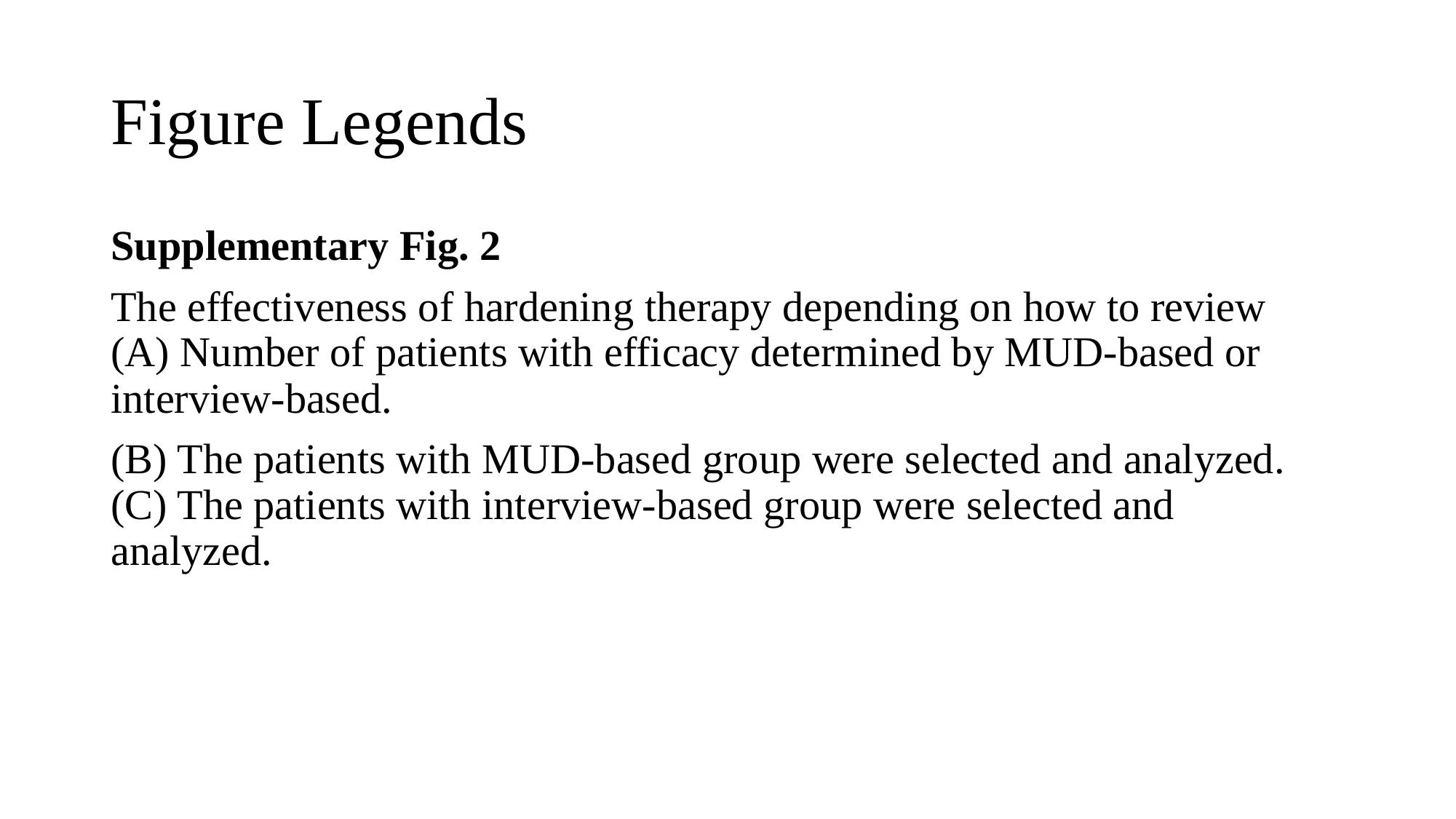

# Figure Legends
Supplementary Fig. 2
The effectiveness of hardening therapy depending on how to review　(A) Number of patients with efficacy determined by MUD-based or interview-based.
(B) The patients with MUD-based group were selected and analyzed. (C) The patients with interview-based group were selected and analyzed.

## Slide 5
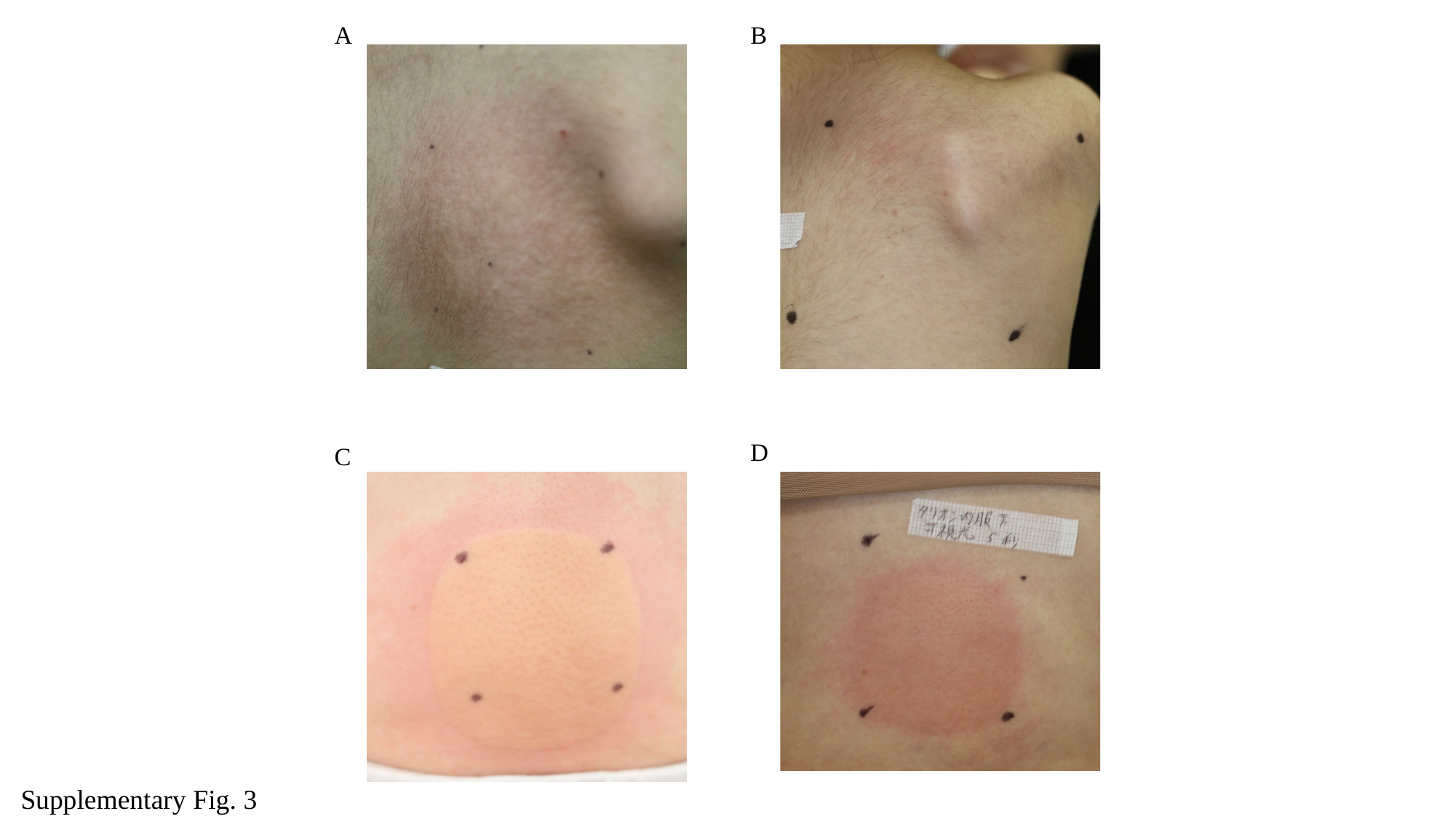

A
B
D
C
Supplementary Fig. 3

## Slide 6
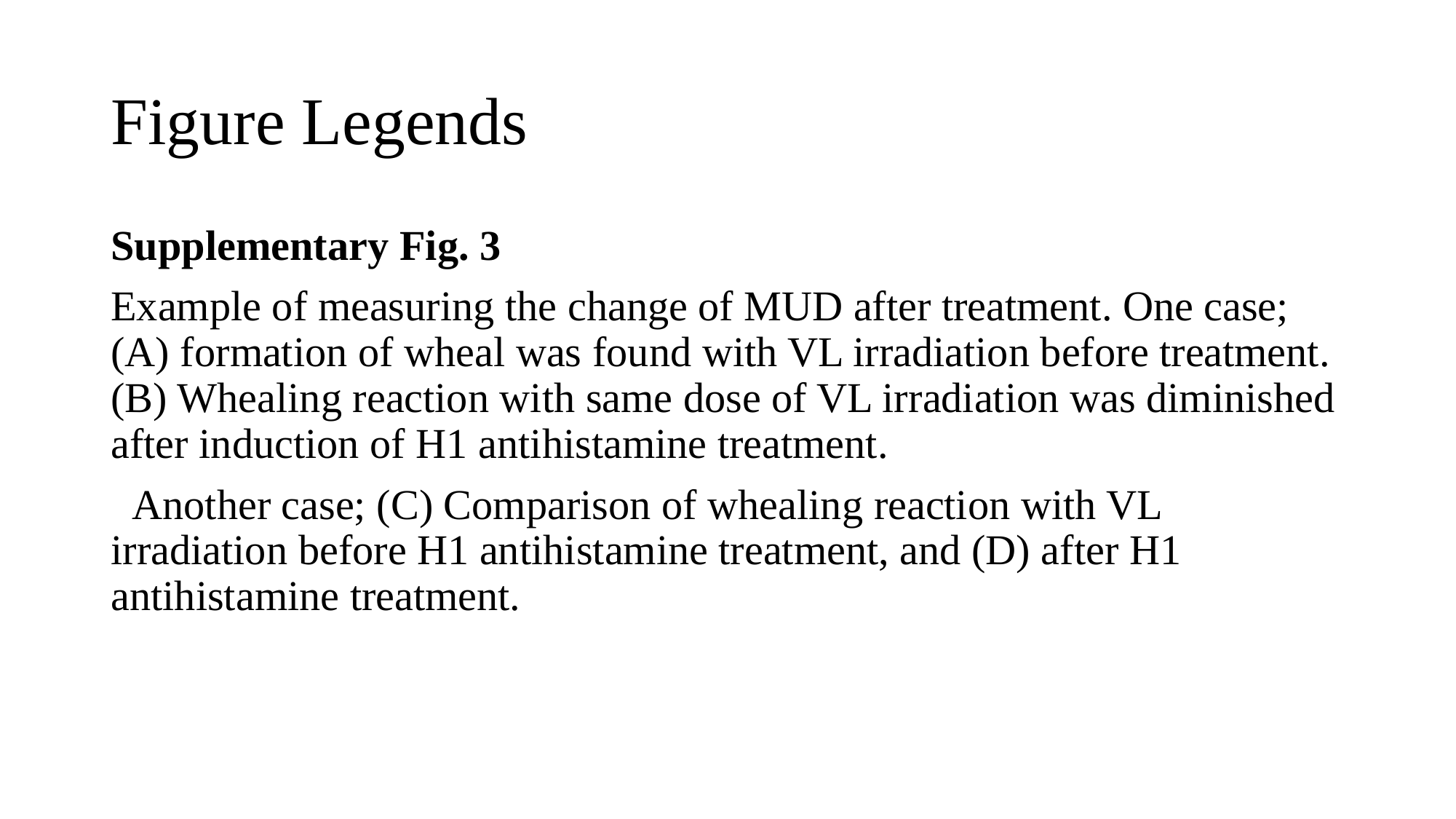

# Figure Legends
Supplementary Fig. 3
Example of measuring the change of MUD after treatment. One case; (A) formation of wheal was found with VL irradiation before treatment. (B) Whealing reaction with same dose of VL irradiation was diminished after induction of H1 antihistamine treatment.
 Another case; (C) Comparison of whealing reaction with VL irradiation before H1 antihistamine treatment, and (D) after H1 antihistamine treatment.
